# Nonverbal Synchrony in Couple Therapy Linked to Clients’ Well-Being and the Therapeutic Alliance

**DOI:** 10.3389/fpsyg.2021.718353

**Published:** 2021-11-11

**Authors:** Petra Nyman-Salonen, Virpi-Liisa Kykyri, Wolfgang Tschacher, Joona Muotka, Anu Tourunen, Markku Penttonen, Jaakko Seikkula

**Affiliations:** ^1^Department of Psychology, University of Jyväskylä, Jyväskylä, Finland; ^2^Faculty of Humanities and Social Sciences, Department of Social Sciences and Philosophy, University of Jyväskylä, Jyväskylä, Finland; ^3^Faculty of Social Sciences/Psychology, University of Tampere, Tampere, Finland; ^4^University Hospital of Psychiatry and Psychotherapy, University of Bern, Bern, Switzerland; ^5^Faculty of Health and Sport Sciences, University of Agder, Kristiansand, Norway

**Keywords:** couple therapy, nonverbal synchrony, motion energy analysis, surrogate synchrony, therapeutic alliance, client well-being

## Abstract

Nonverbal synchrony between individuals has a robust relation to the positive aspects of relationships. In psychotherapy, where talking is the cure, nonverbal synchrony has been related to a positive outcome of therapy and to a stronger therapeutic alliance between therapist and client in dyadic settings. Only a few studies have focused on nonverbal synchrony in multi-actor therapy conversations. Here, we studied the synchrony of head and body movements in couple therapy, with four participants present (spouses and two therapists). We analyzed more than 2000min of couple therapy videos from 11 couple therapy cases using Motion Energy Analysis and a Surrogate Synchrony (SUSY), a procedure used earlier in dyadic psychotherapy settings. SUSY was calculated for all six dyads per session, leading to synchrony computations for 66 different dyads. Significant synchrony occurred in all 29 analyzed sessions and between the majority of dyads. Complex models were used to determine the relations between nonverbal synchrony and the clients’ well-being and all participants’ evaluations of the therapeutic alliance. The clients’ well-being was related to body synchronies in the sessions. Differences were found between the clients’ and therapists’ alliance evaluations: the clients’ alliance evaluations were related to synchrony between both dyads of opposite gender, whereas the therapists’ alliance evaluations were related to synchrony between dyads of the same gender, but opposite to themselves. With four participants present, our study introduces a new aspect of nonverbal synchrony, since as a dyad synchronizes, the other two participants are observing it. Nonverbal synchrony seems to be as important in couple therapy as in individual psychotherapy, but the presence of multiple participants makes the patterns more complex.

## Introduction

Synchrony is an elementary part of human interaction. Synchrony occurs automatically during conversations as we regulate turn-taking or adjust our nonverbal behaviors, including movement, pitch, and facial expressions, to each other. Synchrony can occur in many domains, from physiological arousal to body movements. In this article, we concentrate solely on the coordination of body movements, hereafter nonverbal synchrony. The tendency to synchronize in human interactions has been studied quite extensively using different research methods, including conceptualizations and computations (Delacherche et al., 2012; for a review, see Vicaria and Dickens, 2016).

Even though research methods and computations vary, nonverbal synchrony has generally been related to positive aspects of the interpersonal relationship. Synchrony increases when participants like each other (Kämpf et al., 2018), are in rapport (Sharpley et al., 2001; Lakin and Chartrand, 2003), have a goal to affiliate with each other (Lakin and Chartrand, 2003), have an incidental feeling of similarity (Guéguen and Martin, 2009), and even during self-disclosure (Vacharkulksemsuk and Fredrickson, 2012). Nonverbal synchrony generates feelings of closeness, similarity, and entitativity and a feeling that the interaction is proceeding smoothly (Vicaria and Dickens, 2016). Nonverbal synchrony leads to affiliation (Hove and Risen, 2009), increases positive affect (Tschacher et al., 2014; Mogan et al., 2017; Galbusera et al., 2019), and even affects self-esteem (Lumsden et al., 2014).

On a social level, nonverbal synchrony enhances social bonding and contributes to a prosocial orientation (Mogan et al., 2017), making participants work better together on a joint task (Valdesolo et al., 2010) and increasing cooperation while diminishing self-advantage behavior (Wiltermuth and Heath, 2009). Some situations decrease nonverbal synchrony between interacting partners: during arguments (Paxton and Dale, 2013), interactions with a tardy partner (Miles et al., 2010), or interactions with an out-group member (Yabar et al., 2006; Bourgeois and Hess, 2008). Interestingly, people tend to synchronize more with their next interaction partner after having experienced exclusion from the previous one (Lakin et al., 2008).

What is the essence of nonverbal synchrony? According to the Russian-doll model of empathy (de Waal, 2007; de Waal and Preston, 2017), nonverbal synchrony can be understood as a bottom-up process of empathy, where synchronizing to the other’s movements makes one implicitly understand the other one better. Nonverbal synchrony helps participants become more emotionally attuned to each other (Stel and Vonk, 2010). Empathic persons tend to synchronize better with others (Sonnby-Borgström, 2002; Finset and Ørnes, 2017). This affective empathy precedes cognitive empathy—that is, the ability to perspective-taking (de Waal, 2007). But nonverbal synchrony has also been reported to relate to cognitive empathy, it has been found to enhance the ability to reason about another person’s mind (Baimel et al., 2015) by reducing the egocentric perspective, thus helping to connect with others (Miles et al., 2010). Nonverbal synchrony occurs in triads as well, and nonverbal synchrony has been suggested as complementary in situations where there is a lack of synchrony or similarity in other modes of interaction, such as language style (Dale et al., 2020).

In the context of psychotherapy, nonverbal synchrony has been proposed as a marker of therapeutic alliance (Koole and Tschacher, 2016). The theoretical framework called the In-Synch model describes how nonverbal synchrony is related to the therapeutic alliance (Koole and Tschacher, 2016). According to the framework, nonverbal synchrony establishes a link between therapist and client at different levels of coupling, ranging from behavioral to physiological; and the more synchrony there is between client and therapist, the better the alliance. According to the model, nonverbal synchrony also builds the foundation for the co-regulation of emotions during therapy, which in turn facilitates the development of the client’s emotion regulation skills. This link between nonverbal synchrony and emotional regulation has been investigated in children: nonverbal synchrony between infant and caregiver predicts more self-regulative skills and better emotional regulation and even empathy in older children (Feldman, 2007). It seems thus plausible that this link could sustain even into adulthood and be at play in the context of psychotherapy.

Empirical evidence shows that more synchrony leads to better outcomes—that is, the clients having fewer symptoms at the end of therapy—and to a stronger therapeutic alliance—that is, a better quality of the relationship better between client and therapist (Ramseyer and Tschacher, 2011). Interestingly, head movement synchrony, which consists mainly of conversational movements related to speaking and listening (for example, nodding), has been related to the global outcome of therapy, whereas body movement synchrony has been related to the alliance in the sessions (Ramseyer and Tschacher, 2014). Nonverbal synchrony has been put forward by other researchers as a process variable influencing the outcome of psychotherapy (Prinz et al., 2021).

High synchrony between therapist and client has not always been found to be beneficial. A high level of synchrony between the therapist and client at the beginning of therapy has been related to poor therapy outcomes (Paulick et al., 2018a), and high synchrony was observed in sessions that were marked with little progress (Ramseyer, 2020). In other contexts, synchrony was found to blur self-other boundaries (Paladino et al., 2010; Wiltermuth, 2012), and to impede self-regulation of affect (Galbusera et al., 2019). Research on attachment styles has found that more securely attached persons may synchronize less with others (Feniger-Schaal et al., 2016). These are factors that seem important in the context of psychotherapy.

Lutz et al. (2020) suggested that it is important for the therapist to be able not to synchronize with clients at the beginning of therapy, because synchronizing could strengthen the client’s negative interpersonal patterns that they bring with them to the therapy. They found that low levels of synchrony in the early stages of therapy were related to earlier improvements in interpersonal change patterns (Lutz et al., 2020). But low levels of synchrony at the beginning of therapy have also been related to client dropout, with a medium level of nonverbal synchrony suggested to be most beneficial (Paulick et al., 2018a).

Client characteristics related to nonverbal synchrony have been studied. Depressed clients were found to be less in synchrony with others (Altmann et al., 2021) as well as clients with social anxiety disorder (Asher et al., 2020). Depressed clients have been found to be less involved in nonverbal synchrony at the beginning of therapy compared to anxious clients, but at the end of therapy there were no differences between depressed or anxious clients (Paulick et al., 2018b). Clients with social anxiety disorders who were involved in a high amount of nonverbal synchrony in the early stages of therapy had fewer interpersonal problems and evaluated the therapeutic alliance more positively at the end of therapy (Altmann et al., 2020). But the results on nonverbal synchrony are somewhat inconsistent and possibly due to differences in synchrony computations, and choice of parameters, as well as different research contexts, client variables, and therapist factors.

More nonverbal synchrony was found in cognitive behavioral therapy, especially in the automated version, than in manualized psychodynamic therapy (Altmann et al., 2020). Prinz et al. (2021) studied whether specific therapeutic strategies were related to nonverbal synchronies in the session and found that nonverbal synchrony was associated with higher mastery (the therapist’s ability to assist the client to cope with past situations) but with less resource activation (the clients becoming acquainted with their own positive and healthy potential, characteristics, abilities, and motivation *via* therapist interventions). Nonverbal synchrony was not associated with problem actuation (the activation of avoided experiences and behavior guided by the therapist) or motivational clarification (the therapist’s ability to guide the client through a process of exploration to gain insight into needs and motives). It is fair to say that research on nonverbal synchrony in psychotherapy is still quite novel, and only some aspects of the effect of nonverbal synchrony on the psychotherapy process have been studied.

Many studies have replicated the finding that nonverbal synchrony occurs above chance level in psychotherapy (Ramseyer and Tschacher, 2011, 2014; Paulick et al., 2018a; Ramseyer, 2020; Prinz et al., 2021). As it seems to be a quite robust phenomenon, this suggests that nonverbal synchrony has an important role in psychotherapy. Nonverbal synchrony can be considered as a marker of the quality of the relationship between therapist and client. One proposition might be that, in accordance with the In-Sync model (Koole and Tschacher, 2016), high movement synchrony reflects a joint effort and mutual adaptation to each other, whereas low synchrony may show either complementary behavior (for instance, soothing as the other one is in distress) or disengagement from the relationship.

Even though nonverbal synchrony in psychotherapy has become a growing research area, nonverbal synchrony in couple therapy is still unexamined. In couple therapy, research on nonverbal synchrony is more complex given the presence of multiple participants and relationships. There is the relationship between the therapist and each spouse, and the relationship between the spouses (allegiance), and in the cases studied here, also the relationship between the therapists.

Here, we explored dyadic patterns of nonverbal synchrony in couple therapy. The data originated from a research project (Seikkula et al., 2015, 2018) that studied the synchrony of autonomous nervous system responses of participants in couple therapy, in which all participants wore equipment to record their responses in some of the sessions analyzed.

Research on nonverbal synchrony between romantic couples is sparse. Synchrony of immediacy behaviors (that regulate psychological distance/intimacy) between spouses has been reported to be more prevalent in satisfied couples (Julien et al., 2000). Synchrony between spouses has been found to lead to feelings of closeness and sexual desire (Sharon-David et al., 2019). Interestingly, couples did not synchronize more rapidly to each other compared to unfamiliar dyads, but both spouses evaluated the onset of synchrony more similarly than unfamiliar dyads, and this was true especially when the couple had evaluated their everyday interactions to be of good quality (Preissmann et al., 2016).

Research on nonverbal synchrony between the therapist and the couple is even more scarce. One study investigated body movements, but not synchrony, in couple therapy. Therapeutic alliance was related to predictable and recurring patterns of bodily movements (i.e., shifting of postures, leaning toward each other) between the couple and the therapist (de Roten et al., 1999). Previously, a case study we conducted found that there was a lot of nonverbal synchrony between the two therapists working together, and synchrony between the therapists was especially notable in sessions that followed sessions with weaker alliance evaluations (Nyman-Salonen et al., 2021). Nonverbal synchrony between therapists was suggested to be an embodied and implicit means of strengthening the therapeutic alliance. In a microanalytic discursive study on alliance formations in couple therapy, we found that the therapist who was listening to the conversation synchronized nonverbally with the client who was not involved in the conversation, which could signal an embodied alliance formation between the listeners (Kykyri et al., 2019). The context of couple therapy brings forth a new aspect of nonverbal synchrony: if two participants are synchronized, there is always someone who is watching the synchrony but not participating in it, who might still be affected by it.

Even though couple therapy is an ecologically valid naturalistic context for studying nonverbal synchrony, causal inferences cannot be made due to the many confounding variables that might affect synchrony and the way it is felt or interpreted by each participant. Therapists and the couple have different roles within the situation; the therapists are in their professional roles, acting accordingly, and are highly familiarized with the context. To the clients seeking help because of issues in their relationships, couple therapy may be a novel situation that could also be threatening. Further, the couple have their own relationship history, which makes them react to each other in predisposed ways. Moreover, in couple therapy, both spouses react to the situation separately but also as a part of their couple system. In couple therapy, there can be hidden variables or agendas that presumably affect how the participants synchronize with each other. We aimed to study whether nonverbal synchrony in couple therapy occurred between all the possible dyads and whether it was related to the clients’ well-being, the therapeutic alliance in the sessions, and to the outcome of therapy.

A strong alliance has been related to a positive outcome in individual psychotherapy (Horvath and Symonds, 1991; Lambert and Barley, 2001), but also in couple therapy (Bourgeois et al., 1990; Johnson and Talitman, 1997; Anker et al., 2010). However, the relationship between therapeutic alliance and outcome in couple therapy is not as straightforward as in individual psychotherapy (Friedlander et al., 2011, 2018), since multiple different alliances can influence the relationship. There is an alliance between one of the spouses and the therapist, the alliance between the other spouse and the therapist, and the alliance between the couple as a system and the therapist (Pinsof, 1995; Mamodhoussen et al., 2005). There is also a relationship between the spouses that might have a bearing on the therapeutic alliance (Pinsof, 1995).

Different factors influence the relationship between alliance and outcome in couple therapy. For instance, the relationship between alliance and outcome becomes stronger when both spouses agree on the strength of the alliance (Pinsof and Catherall, 1986; Symonds and Horvath, 2004). Even gender differences have been found concerning the relationship between alliance and outcome. The alliance evaluated by the male clients has been reported to be more strongly related to the outcome than the female client’s evaluations (Bourgeois et al., 1990; Symonds and Horvath, 2004; Anker et al., 2010; Glebova et al., 2011). But if women rate their partner’s alliance with the therapist more positively, a successful outcome is more likely; and when the male client evaluates the alliance to be stronger than what the female clients evaluate, marital distress decreases (Knobloch-Fedders et al., 2007). Tentatively speaking, it seems important for both spouses that the male partner’s evaluations of the alliance is positive.

Nonverbal synchrony could also be a method for studying alliance in couple therapy, as it has been suggested to be a marker of therapeutic alliance in individual therapy (Koole and Tschacher, 2016). Different methods have been used to quantify nonverbal body movement synchrony. In this study, we used Motion Energy Analysis, hereafter MEA (Ramseyer and Tschacher, 2011), because it is the method that has been used the most in research on nonverbal synchrony in individual psychotherapy; however, it has not been used in couple therapy. Our research aim was to explore whether nonverbal synchrony between participants in couple therapy was related to the clients’ well-being, therapeutic alliance and therapy outcome.

### Research Questions

RQ 1: We hypothesized that nonverbal synchrony of head and body movements occurred above chance level in the whole dataset. More specifically, there would be significant synchrony between all dyads in all sessions. We were also interested in whether there was a mean difference between head and body synchrony between three different types of dyads (client–client, client–therapist, and therapist–therapist).

RQ 2: We hypothesized that the well-being of the clients, the alliance, and the outcome of therapy would be related to the nonverbal synchrony patterns in the session.

## Materials and Methods

### Design and Participants

The couple therapy data were collected in the research project Relational Mind in Events of Change in Multi-actor Dialogues, which took place at the Psychotherapy Training and Research Centre of the Department of Psychology at the University of Jyväskylä (Seikkula et al., 2015). At the facility, it is common practice for therapists to work in dyads with couples. The research project studied embodied attunement between the participants in couple therapy. The therapy was not manualized but was influenced by dialogical therapy.

The overall Relational Mind data consisted of 12 couple therapy cases, of which 11 consisted of man and woman. For all therapies, two therapists were present. Ten therapists worked with the couples; that is, many of the therapists worked on more than one couple therapy case. Normally, the therapist dyads varied, but one dyad worked on two cases. The therapists were between 31 and 64years old, mainly with a degree in family therapy (7 out of 10 therapists). All but one therapist had over 10years of experience from clinical work. Six of the 10 therapists were female.

The therapy sessions were recorded using six cameras: one camera focused on each participant’s face and one camera recorded the full bodies of the two therapists and the couple. The couple and the therapists were seated in chairs around a round table: The clients sat next to each other, and the two therapists sat next to each other on the opposite side of the table.

Because of the research group’s interest in autonomous nervous system responses, all participants’ autonomous nervous system reactions were usually recorded in the second and sixth session. In these measurement sessions, heart rate monitors were attached to the chest, two skin conductance electrodes were attached to the palm of the nondominant hand, and a respiration rate belt was fastened around the lower chest. The skin conductance electrodes were attached to the chair in which the participant sat and thus restricted the movement of the non-dominant arm to a range of approximately 25cm from the chair.

The well-being (outcome) and alliance were assessed using the ultra-brief forms of the Outcome Rating Scale (ORS) and the Session Rating Scale (SRS; Duncan et al., 2003; Miller et al., 2003), and the outcome with the Clinical Outcomes in Routine Evaluation – Outcome Measure (CORE-OM) questionnaire (Barkham et al., 2001; Evans et al., 2002). SRS and ORS have been used in the context of couple therapy (Anker et al., 2010; Kuhlman et al., 2013). ORS is a short outcome measurement that measures the well-being of the clients. It was given to both clients before each session; the SRS measures the session-level alliance and was given to both clients and therapists after each session. Both the SRS and ORS are visual analogue self-report measures, and the participants marked their answer to the question by making a cross on a 10-cm long line. The results were converted to numbers by measuring the place of the cross, and then numbered using 0 (left) to 10 (right), making a Likert-type scale. The ultra-brief form of ORS measures well-being with four items: general sense of well-being (Overall), personal well-being (Individually), well-being in relation to one’s family and close relationships (Interpersonally), and well-being in relation to one’s work or school and friendships (Socially). The SRS has four items depicting four different aspects of alliance. The “Relationship” scale comprises the item “I felt/did not feel heard, understood, and respected,” and the “Goals and Topics” scale comprises “We worked on or talked about/did not work or talk about what I wanted to work on or talk about.” The “Approach or Method” scale requires rating the session based on the item “The therapist’s approach is/is not a good fit for me.” The fourth question rates the “Overall session” with the item “There was something missing in the session today” vs. “Overall, today’s session was right for me.”

The outcome of the therapy was assessed with the CORE-OM questionnaire (Barkham et al., 2001; Evans et al., 2002), administered to the clients in the first session, after the last session, and at a follow-up after 6months. CORE-OM is a standardized brief self-report instrument for evaluating change in psychotherapy. It covers four domains: subjective well-being, problems (depression, anxiety, physical aspects, effects of trauma), functioning (close relationships, general functioning, and social aspects), and risk (to self and to others; Barkham et al., 2001; Evans et al., 2002).

The research procedure was approved by the University of Jyväskylä Ethical Committee. All participants gave their written informed consent to participate in the research project.

### Data Selection

For the movement analysis, one couple therapy case was omitted, since one of the spouses suffered from obsessive movement patterns, which affected the data. Videos from 11 couple therapy cases were used. Of the 11 couples, seven were married (one registered partnership), three were living together, and one couple lived separately. All of the couples had been together for over a year and almost all of them had been together for several years. The mean age of the female clients was 41 (range=27–54), and the mean age of the male clients was 44 (range=34 to 61). Mean psychotherapy duration per couple was six sessions (*M*=6.27, Mdn=6), and duration varied between cases (Min=4, Max=10).

The inclusion criteria of the therapy sessions for movement analysis were done based on the parameters required by MEA: the lighting needed to be stationary and the video screen needed to show all participants’ full bodies. The participants needed to be seated at all times, and all regions of interest (head and body) needed to be visible in the video at all times. For MEA, videos showing the full bodies of the participants in a split-screen format were used.

From a pool of 69 videos, 29 met these criteria, which indicates one to three sessions per case (*M*=2.6, *SD*=0.7, Mdn=3). Out of the qualified videos, 17 were from measurement sessions, and 12 were from regular sessions.^1^ The videos were converted to QuickTime format, edited to 10 frames per second, cut from the beginning to the point where all participants sat in their chairs, and cut at the end when participants began taking out their calendars to schedule the next meeting. This resulted in sessions that lasted, on average, 79min (*SD*=8.29min, Min=52min, Max=90min).

### Analysis Procedure

All selected sessions were analyzed with MEA (Ramseyer and Tschacher, 2011). MEA is an automated computer program designed to quantify movements from video recordings. Motion energy is defined as the amount of gray-scale pixel changes occurring between consecutive video-frames. The changes are calculated within a region of interest (ROI) that can be manually defined on the video screen. Given that the context was couple therapy with two therapists present, eight ROIs were defined: the head and the body of each participant separately. Preprocessing of the data was first done on the basis of the videos: The ROIs of each participant’s head and body were checked manually in each video before the extraction of the data to guarantee that no overlapping of movement between the different ROIs occurred (as the full-body videos of one dyad were filmed from behind the other dyad, sometimes one participant of a dyad leaned forward and visually entered the ROI of the other dyad, resulting in erroneous data). MEA then generated a time series of pixel changes for all defined ROIs. Preprocessing at the MEA level was performed by setting the threshold for recording of pixel changes at a value of 15, which is the default of this procedure. Thus, all pixel changes inside a ROI less than 15 were considered as video noise and disregarded. Additionally, the spurious peaks at the beginning of MEA records, which however last only for less than 1s, were deleted.

After obtaining the raw data from the MEA, movement synchrony between different ROIs was computed using the Surrogate Synchrony (SUSY) procedure (Tschacher and Haken, 2019; for a web-based app see http://www.embodiment.ch). SUSY allows dyadic synchrony to be computed: head and movement synchrony for six dyads (client 1–client 2, therapist 1–therapist 2, and all four client–therapist dyads) was calculated. SUSY divided the time series of the MEA individual movement raw data into segments of 30s. In each segment, all the cross-correlations were calculated up to time lags of +/− 5s by shifting one of the time series stepwise (in 0.1s steps because of the sampling rate of 10 frames/s) in relation to the other one. The cross-correlations were standardized using Fisher’s *Z*, which were then aggregated to a mean *Z* value of nonverbal synchrony for all lags separately in each segment. The mean *Z* values of all segments were averaged, resulting in a mean *Z* value of nonverbal synchrony for the whole therapy session for each dyad and synchrony type (head and body). SUSY calculates the mean *Z* synchrony using both absolute values from the cross-correlations (*Z*_abs_), by converting negative values of cross-correlations into positive ones, and the original positive and negative (thus, ‘non-absolute’) values of the cross-correlations (*Z*_noabs_). Using non-absolute values (*Z*_noabs_) enables distinguishing between in-phase synchrony (i.e., both participants’ movements are positively correlated) and anti-phase synchrony (i.e., both participants’ movements are negatively correlated: when one is moving more the other one is moving less). Both absolute and non-absolute cross-correlations and in-phase and anti-phase synchrony of datasets have been interpreted by Tschacher and Meier (2020) and Coutinho et al. (2021).

To investigate whether synchrony occurred above chance level, surrogate datasets were created by shuffling the segments of the original data from the two time series, aligning segments that never occurred at the same time. Many surrogate datasets can be generated from the data of a session, for example, in a 50-min session containing 100 segments, 100×99=9,900 surrogate datasets. A value of the pseudo synchrony of each surrogate dataset was then computed in the same way as the synchrony computations described above. Lastly, the empirically obtained synchrony calculations were standardized using pseudo-synchronies by comparing the mean value of the surrogate data to the same value of the empirically collected synchrony, giving the effect size for each dyadic head and body synchrony in the session. The effect size was obtained for both absolute values (ES_abs_) and non-absolute values (ES_noabs_). We used the non-absolute effect sizes (ES_noabs_) for all statistical calculations, since they allow for the distinction of in-phase and anti-phase synchrony.

The head and body synchrony effect sizes (ES_noabs_) of each of the six dyads (client 1–client 2, therapist 1–therapist 2, female client–female therapist, female client–male therapist, male client–female therapist, male client–male therapist) were obtained from all sessions, resulting in 12 dyadic nonverbal synchrony effect sizes (ES_noabs_) per session. Contrary to the earlier research, we used the movement data from the whole session for the synchrony computations, resulting in a more valid value of nonverbal synchrony between participants. We used the gender of the participants to distinguish between the four participants in each situation.

The objective for using SUSY was twofold: First, it is the synchrony computation method that has been used the most in psychotherapy research. Second, as the context is psychotherapy, in which the dialogue, and the embodied responses of the participants unfold in seconds, it was important to use a method that enables synchrony computation using time lags of several seconds as the time unit. This kind of synchrony calculation depicts the movement interaction between participants in the therapy setting in a ecologically valid way, reflecting the embodied responsiveness between participants.

### Statistical Analyses

#### The Data

The data came from 11 couple therapy cases, 1–3 sessions from each case were analyzed. Intraclass correlations (ICCs) were computed to determine whether the data were indeed hierarchical (significant amount of the total variance of the dyadic nonverbal synchrony effect sizes was between cases). The ICCs were calculated in MPlus version 8.4 using two-level models (level 1 within, level 2 between) with Maximum Likelihood with robust standard errors (MLR) as estimator. Six models with two variables in each model were calculated.

Due to the hierarchical data set, and thus the non-independence of the nonverbal synchrony effect sizes (which were nested within cases), complex models were used for the majority of the statistical analyses. Complex models have been developed for analyzing clustered data (Muthén and Satorra, 1995). Complex models take into account the clustered sample by correcting the standard errors using a sandwich estimator, thus giving more reliable values of *p*. The small dataset and the small number of clusters restricted the number of estimated parameters in one model. Thus, several one-level complex models were used for estimating correlational relations and for comparing means. The number of models is specified below for each computation. The models were all computed using MLR as estimator, and case was used as the cluster variable. All models were saturated, meaning that all degrees of freedom were used, and thus fitted the data perfectly. All complex models were computed using MPlus version 8.4.

#### Individual Movements

We studied the individual movements of each participant to gain a full picture of the data used for the nonverbal synchrony calculations. The individual amount of movement of head and body per participant in each session was obtained with MEA, and the data were organized according to gender. The amount of movement was adjusted to the length of the session, providing comparable values. Six complex models were calculated to estimate the difference between how much the participants moved their head and body. The following three pairs were compared: female client vs. male client (head and body in separate models), female therapist vs. male therapist (head and body in separate models), and the mean of both clients vs. the mean of both therapists (head and body in separate models). The nonverbal synchrony effect sizes were designated as dependent variables and their means were compared.

#### Measurement Sessions

To assess potential influence of wearing measurement equipment on the participants’ movement patterns, three complex models were used to estimate the differences in how much the participants moved in regular vs. measurement sessions: The first model included the mean of all participants’ head movements, the second model included the mean of all participants’ body movements, and the third model included the mean of all participants’ head and body movements. Session type was designated as the independent variable and movement as the dependent variable. As for the comparison of nonverbal synchronies in regular sessions vs. measurement sessions two complex models (head and body separately) were calculated for each dyad type (client 1–client 2, therapist 1–therapist 2, client–therapist) with session type as independent variable and synchrony as the dependent variable. For the client–therapist dyad, a mean of nonverbal synchrony of all four possible dyads (female client–female therapist, female client–male therapist, male client–female therapist, and male client–male therapist) was used.

#### Nonverbal Synchrony (RQ 1)

To study whether nonverbal synchrony in the whole data set occurred above chance level as expected, Cohen’s *d* was calculated according to the procedure described by Tschacher and Meier (2020). The difference between the mean *Z*_noabs_ of all *N* sessions and the mean *Z*_noabs-pseudo_ of the surrogate dataset of all *N* sessions was divided by the standard deviation of the *Z*_noabs-pseudo_ for the surrogate data set. Cohen’s *d* is thus an effect size at the level of all *N* sessions.

To calculate whether the head and body synchrony between each of the six dyads was significant in each session, the effect sizes (ES_noabs_) of each dyadic synchrony value (*N*=12) per session were computed using one-sample *t*-tests.

The means of head and body synchrony between the three different types of dyads (client–client, client–therapist, therapist–therapist) were compared using six complex models: (1) client–client vs. client–therapist head synchrony, (2) client–client vs. client–therapist body synchrony, (3) client–client vs. therapist–therapist head synchrony, (4) client–client vs. therapist–therapist body synchrony, (5) therapist–therapist vs. client–therapist head synchrony, and (6) therapist–therapist vs. client–therapist body synchrony. The synchrony value for the client–therapist dyad consisted of the mean of nonverbal synchrony of all the four different dyads (female client–female therapist, female client–male therapist, male client–female therapist, male client–male therapist). The nonverbal synchrony effect sizes were designated as dependent variables.

#### Clients’ Well-Being and Nonverbal Synchrony

The relationship between the clients’ self-reported well-being (ORS) and nonverbal synchrony was calculated with complex models. Six complex models were used for both female and male clients to calculate the relationship between the client’s ORS and all six dyadic (client 1–client 2, therapist 1–therapist 2, female client–female therapist, female client–male therapist, male client–female therapist, male client–male therapist) head and body synchronies. Two additional complex models, one per client, were calculated to find out the relationship between ORS and nonverbal synchrony between one of the spouses and both therapists (for this the mean synchrony between female client–therapist 1 and female client–therapist 2, as well as the mean synchrony between male client–therapist 1 and male client–therapist 2 was used). In these eight aforementioned models, both ORS and nonverbal synchrony were designated as dependent variables.

To study the relation between the mean of both clients’ ORS and the mean of all head and body synchronies two complex models were calculated, where ORS was designated as the independent variable and synchrony as the dependent variable. One model included the mean of both clients’ ORS and the mean of all head synchronies, and the other model included the mean of both clients’ ORS and the mean of all body synchronies.

To calculate whether taking part in synchrony or observing had any impact on the relationship between ORS and nonverbal synchrony two new aggregated variables per client were computed: one variable for the mean of all dyadic synchronies in which the client participated, and another variable for the mean of the nonverbal synchronies that the client observed. The new variables were used in one complex model per client, where all variables were treated as dependent variables.

#### Therapeutic Alliance and Nonverbal Synchrony

The relationship between all participants’ evaluations of the alliance (SRS) and the non-absolute effect sizes of nonverbal synchronies was calculated almost identically as the relationship between ORS and nonverbal synchrony: First, the relationship between all participants’ SRS evaluations and the nonverbal synchrony of the six dyads were calculated separately for each participant. One complex model included the SRS evaluation of one participant and head and body synchrony of one dyad, thus six complex models for each participant were calculated (in which all variables were designated as dependent variables). Second, the relation between the mean of both clients’ SRS and the mean of both therapists’ SRS with the mean of all participants’ head synchrony (one complex model) and body synchrony (one complex model). The two mean SRSs were designated as independent variables and the mean of synchrony as the dependent variable. Third, we studied the relationship between each participant’s SRS and the nonverbal synchronies in which they participated or observed. One complex model per participant was calculated with the SRS and the two new variables (participated, observed) as dependent variables.

#### Outcome and Nonverbal Synchrony

CORE-OM was filled by all participants at the beginning and end of therapy, and after a 6month follow up. The relationship between the outcome (change in CORE-OM) and nonverbal synchrony was calculated using one aggregated head synchrony value and one aggregated body synchrony value for each case and dyad. The aggregation was done because of CORE-OM only giving three change values (beginning to end, beginning to 6months, end to 6months) for each client to represent the whole therapy process. There was unfortunately a large amount of missing data in the CORE-OM because clients failed to return their questionnaires, which resulted in an extremely small sample size (*N*=6). Spearman’s rank order correlations were used (because of outliers in nonverbal synchrony values). Bootstrapping was not used because of the small *N* possibly distorting the bootstrapped sample. The calculations were performed using IBM SPSS Statistics version 26. First Spearman’s rank order correlations were computed for the six dyads’ head and body synchronies and the basic CORE-OM change scores (beginning to end, beginning to 6months and end to 6months) for each client separately. Second, a mean of all the participants’ head synchronies and a mean of all body synchronies were correlated with the participants’ CORE-OM change scores. Third, the mean of both clients’ CORE-OM scores was correlated with all the dyadic nonverbal synchronies.

## Results

### The Data

First, we explored the basic characters of the data, and computed ICCs of all dyadic nonverbal synchronies (ES_noabs_) to establish if the data was hierarchical. After this we studied the individual movement patterns of each participant to get an overview of the data used to compute the dyadic nonverbal synchronies. We investigated the validity of the data, that is, whether wearing measurement equipment affected individual movement patterns or nonverbal synchrony patterns.

### Intraclass Correlations

The ICCs show how many percent of the whole variance is between cases. ICCs were calculated for each dyadic nonverbal synchrony value (ES_noabs_) using 60 two-level models (level 1 within, level 2 between). The majority (58%) of the dyadic nonverbal synchrony effect sizes (ES_noabs_) had significantly more variance between cases than within cases, pointing to the data being hierarchical. All results are provided in Table 1.

**Table 1:** Intraclass correlations of the head and body synchronies for each dyad.

	Client–client	Female client and male therapist	Female client and female therapist	Male client and male therapist	Male client and female therapist	Therapist– therapist
Head synchrony	0.475^*^ p <0.001	0.454^*^ p <0.001	0.430 *p* =0.156	0.251 *p* =0.140	0.511^*^ p =0.009	0.343 *p* =0.072
Body synchrony	0.133 *p* =0.297	0.740^*^ p <0.001	0.329^*^ p =0.015	0.333^*^ p =0.001	0.273 *p* =0.254	0.457^*^ p =0.019

* Significant result.

*N*=29.

### Individual Movement

The amount of individual movement of each participants’ head and body adjusted by the length of the sessions were calculated to understand the data underlying the dyadic nonverbal synchrony patterns. A significant difference was found between all head movement means between all dyads (female vs. male clients, female therapists vs. male therapists, and clients vs. therapists), as shown in Table 2. For individual body movements, the only significant difference was between the mean of both clients’ and the mean of both therapists’ body movements (clients’ *M*=143.36, therapists’ *M*=105.69). No other significant differences were found. All results are provided in Table 2.

**Table 2:** The mean differences between the individual movement between participants.

Dyads	Female client (A) - male client (B)	Female therapist (A) - male therapist (B)	Client (A) - therapist (B)^†^
Head *β*	35.865^*^ p =0.002	30.748^*^ p =0.011	31.218^*^ p =0.001
mean A	105.950	41.426	88.018
mean B	70.086	72.174	56.800
Body *β*	23.731 *p* =0.271	42.655 *p* =0.165	37.664^*^ p =0.027
mean A	155.225	84.369	143.360
mean B	131.494	127.024	105.694

* Significant result.

† The client–therapist comparison was performed based on the mean of the movements of both clients and the mean of the movements of both therapists.

### Measurement Sessions

There was no difference between individual movements in the regular vs measurement sessions. No significant differences were found for head movements (*β*=−1.15, *p*=0.91), body movements (*β*=−1.16, *p*=0.95), or all movements (*β*=−1.16, *p*=0.93).

For the nonverbal synchrony, the only significant relation was that there was less therapists’ head synchrony in the measurement sessions (*β*=−0.31, *p*=0.046). All results are provided in Table 3.

**Table 3:** Estimates of the difference between synchrony in regular and measurement sessions.

Dyad	Client–client	Therapist–therapist	Client–therapist^†^
Head synchrony	−0.247 *p* =0.075	−0.309^*^ p =0.046	0.042 *p* =0.672
Body synchrony	0.029 *p* =0.890	−0.088 *p* =0.582	−0.239 *p* =0.071

* Significant result.

† For the client–therapist dyads the mean of the synchrony from the four dyads (female client and female therapist, female client and male therapist, male client and male therapist, male client and female therapist) was calculated.

### Nonverbal Synchrony in the Whole Data and in All Dyads (RQ 1)

We hypothesized that there would be significant dyadic nonverbal synchrony in the whole data set. To obtain the effect size of the overall synchrony of the whole data set, Cohen’s d was calculated based on all the effect sizes of all dyads (6×2×29) in all sessions (*N*=29) by using the method described earlier (Tschacher and Meier, 2020). The effect size for the whole dataset (*d*=1.36) met Cohen’s (1988) convention for a large effect (*d*>0.80).

We also hypothesized that there would be significant nonverbal synchrony (ES_noabs_) in all sessions and between the majority of dyads. Twelve values of nonverbal synchrony per session were obtained, head synchrony for each dyad (*N*=6), and body synchrony for each dyad (*N*=6). For all cases and dyads, this resulted in 348 different synchrony values. However, 32 values were treated as missing because some of the cases were not gendered balanced (in three sessions with a female–female couple, the dyadic nonverbal synchronies in which the male client was included were missing, and in five sessions with male–male therapist dyads, the nonverbal synchronies in which the female therapist was included were missing, since they did not fit into the classification pattern of gender-based dyadic synchronies). We did not omit any synchrony values from the client-client and therapist-therapist dyads. This resulted in 316 synchrony values. Using one-sample *t*-tests on the effect sizes of each dyadic synchrony, the significance of the nonverbal synchronies was calculated for all synchrony values (*N*=316). Of all nonverbal synchrony effect sizes 97% (*N*=307) were significant and 3% were not significant (*N*=9). Out of the effect sizes 189 were positive, indicating in-phase synchrony, and 127 effect sizes were negative, indicating anti-phase synchrony. A summary of the significances is provided in Table 4.

**Table 4:** The amount of significant and not significant nonverbal synchrony per dyad.

Dyads	Client and client		Female client and male therapist		Female client and female therapist		Male client and male therapist		Male client and female therapist		Therapist and therapist	
	Head	Body	Head	Body	Head	Body	Head	Body	Head	Body	Head	Body
**In-phase synchrony**												
Significant	13	9	12	8	18	16	15	16	14	12	29	25
Not significant	0	0	0	1	0	0	0	0	0	1	0	0
**Anti-phase synchrony**												
Significant	13	20	17	20	6	7	9	9	7	8	0	4
Not significant	3	0	0	0	0	1	2	1	0	0	0	0
*n*	29	29	29	29	24	24	26	26	21	21	29	29
missing	0	0	0	0	5	5	3	3	8	8	0	0

Significant *p*<0.05. All in-phase synchronies (N = 189), all anti-phase synchronies (N = 127).

Nonsignificant in-phase synchrony was found for two body movement synchrony effect sizes (of the 189 effect sizes) between two different dyads (female client and male therapist; male client and female therapist) in two different cases. Nonsignificant anti-phase synchrony was found for seven nonverbal synchrony effect sizes (of the 127 effect sizes): five head movement synchronies and two movement body synchronies. Nonsignificant anti-phase head synchrony between clients was found in three different cases and sessions. Anti-phase head movement synchrony between the male client and the male therapist was nonsignificant in two different cases and sessions (one of the sessions also had nonsignificant client–client head synchrony). In one session, anti-phase body movement synchrony was nonsignificant in two dyads: between the male client and the male therapist and between the female client and the female therapist.

**Figure 1:**
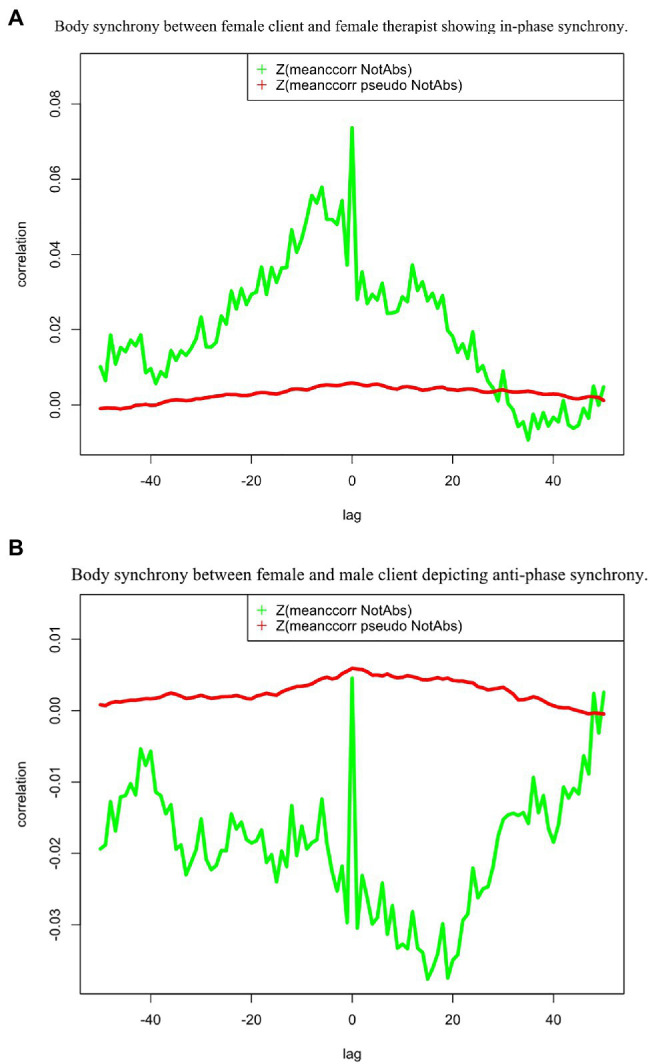
Both plots represent dyadic synchrony from one couple therapy session. The green graph depicts the real nonverbal synchrony cross-correlations as a function of the respective lag. The red graph is the average of all surrogate time series and represents the pseudo-synchronies. The upper panel **(A)** shows body synchrony between the female client and female therapist in one session. The green graph is above the red graph showing significant in-phase synchrony (positive correlations). In the lower panel **(B)** body synchrony between the clients in the same session is depicted. The green graph is below the red pseudo-synchrony graph, showing anti-phase synchrony (negative correlations).

In-phase and anti-phase synchrony can be distinguished by the effect size being positive (in-phase synchrony) or negative (anti-phase synchrony). Figure 1 shows two cross-correlation functions depicting body synchrony between two different dyads in the same session, the first one being in-phase synchrony, and the second showing anti-phase synchrony.

We further assessed potential differences in means between the synchrony of head and body movements (ES_noabs_) between the three different types of dyads (client–client, client–therapist, therapist–therapist). The nonverbal synchrony effect size of the client–therapist dyad was the mean of the four client–therapist dyads. All comparisons were significant except for head movement synchrony between the client–client dyad and the client–therapist dyad (*β*=1.516, *p*=0.248). All estimates and the mean of synchrony of each dyad type can be seen in Table 5.

**Table 5:** The differences in the means between head and body synchrony for the three different types of dyads.

Dyads	Client–client (A) vs. client–therapist^†^ (B)	Client–client (A) vs. therapist–therapist (B)	Therapist–therapist (A) vs. client–therapist^†^ (B)
Head *β*	−1.516 *p* =0.248	−7.664^*^ p <0.001	6.149^*^ p <0.001
mean A	−0.665	−0.665	6.999
mean B	0.850	6.999	0.850
Body *β*	−2.106^*^ p =0.038	−5.883^*^ p <0.001	3.778^*^ p <0.001
mean A	−1.438	−1.438	4.445
mean B	0.668	4.445	0.667

* Significant results.

† The client–therapist dyad’s effect sizes (ES_noabs_) were the mean of all client–therapist dyads (female client–female therapist, female client–male therapist, male client–female therapist, male client–male therapist).

*Post hoc* power analysis were computed for the comparisons between the dyads. The client–client and the client–therapist dyad head synchrony reached a *post hoc* power of 0.279 and body synchrony a *post hoc* power of 0.496. The comparison of the client–client and therapist–therapist dyad head synchrony reached a *post hoc* power of 1.000 and body synchrony a *post hoc* power of 0.972. The comparison between the therapist–therapist and client–therapist dyad head synchrony reached a *post hoc* power of 1.000 and body synchrony a *post hoc* power of 0.989.

Out of interest to find out whether the sample size was too small especially when using the mean value for the client–therapist dyad, a *post hoc* power analysis was conducted using MPlus version 8.4 using Monte Carlo simulation with 1,000 replications to test whether the sample size had enough power at the level of an alpha of 0.05. For four out of five of the significant relations, the *post hoc* power was above 0.9. For the nonsignificant comparison (client–client vs. client–therapist head synchrony), the *post hoc* power was 0.279 for head synchrony, and 0.496 for body synchrony. All *post hoc* powers are shown in Table 5.

### Clients’ Well-Being and Nonverbal Synchrony

The relationships between the clients’ well-being (ORS) and head and body synchronies were calculated using several complex models (see method section). ORS was administered to the clients before each session, which meant that we tested whether the well-being of the clients at the beginning of the session was related to the synchronies later on in the session. The mean of both clients’ ORS was significantly related to the mean of all body synchronies (ES_noabs_) across the whole data (*β*=0.537, *p*=0.004), whereas the relationship of ORS to the mean of head synchronies (ES_noabs_) was not significant (*β*=0.276, *p*=0.280). All relationships between the female and male client’s ORS and the dyadic synchronies (ES_noabs_) are shown in Table 6.

**Table 6:** The relations between the clients’ Outcome Rating Scale (ORS) and head and body synchronies (ES_noabs_).

Dyads	Client and client		Female client and male therapist		Female client and female therapist		Male client and male therapist		Male client and female therapist		Therapist and therapist	
	Head	Body	Head	Body	Head	Body	Head	Body	Head	Body	Head	Body
Female client ORS *β* (*p*)	−0.182 (0.554)	−0.13 (0.954)	0.259 (0.110)	0.517^*^ (0.003)	0.057 (0.790)	−0.037 (0.858)	0.207 (0.267)	0.072 (0.549)	0.508^*^ (0.006)	0.543^*^ (0.000)	0.130 (0.450)	0.234 (0.325)
Male client ORS *β* (*p*)	−0.125 (0.603)	−0.30 (0.877)	−0.139 (0.636)	0.359 (0.118)	0.461^*^ (0.015)	0.161 (0.373)	0.098 (0.626)	0.086 (0.491)	0.418^*^ (0.038)	0.673^*^ (0.000)	0.366^*^ (0.001)	0.522^*^ (0.000)

* Significant result.

Complex models were computed to determine whether there was a difference in the relationships between ORS and the synchronies in which a client participated or observed. For ORS, no significant relations were found for either of the clients concerning nonverbal synchronies in which they participated or observed (female clients participated *β*=0.407, *p*=0.063, or observed *β*=0.073, *p*=0.785; male clients participated in *β*=0.240, *p*=0.081 or observed *β*=0.372, *p*=0.050).

### Alliance and Nonverbal Synchrony

**Figure 2:**
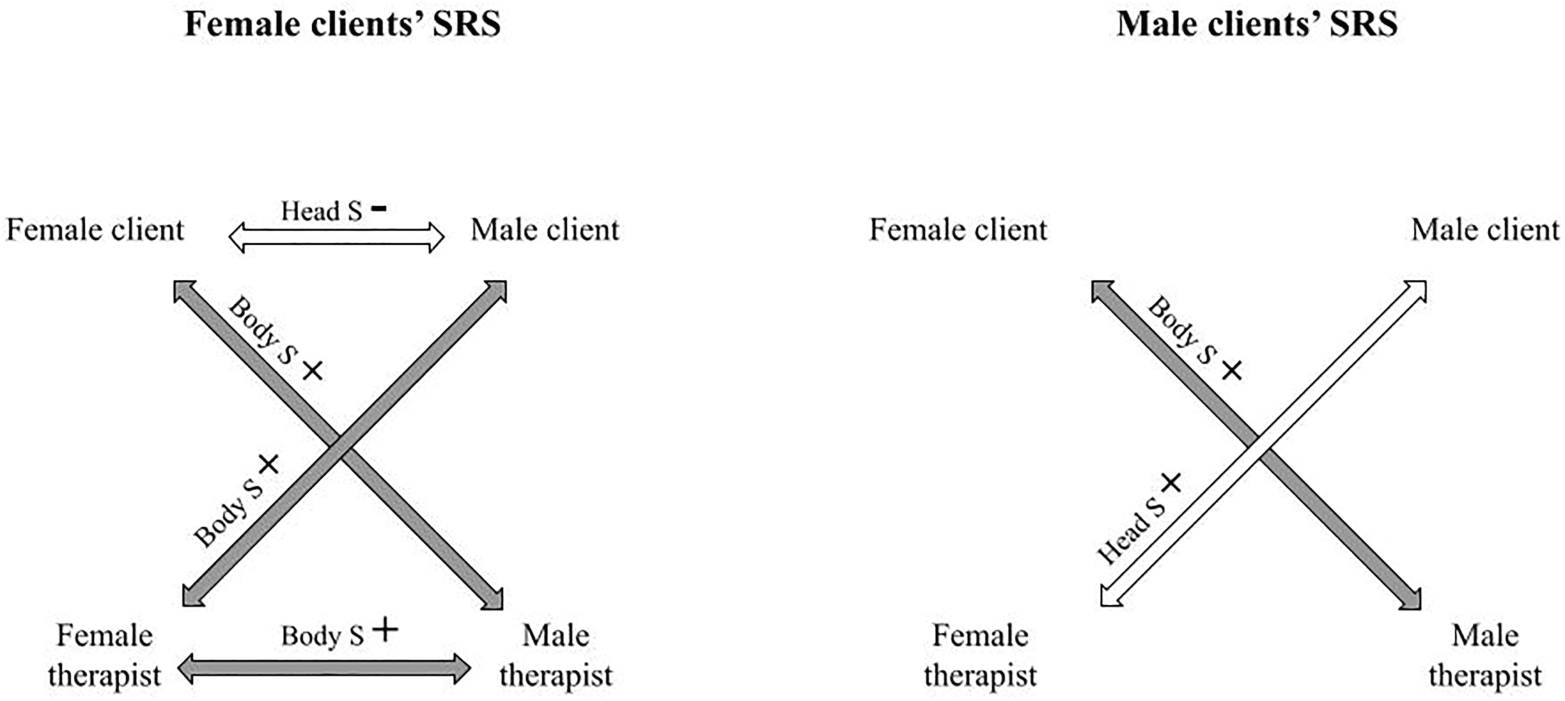
The relationship between the clients’ alliance evaluations and the dyadic head and body synchronies.

**Figure 3:**
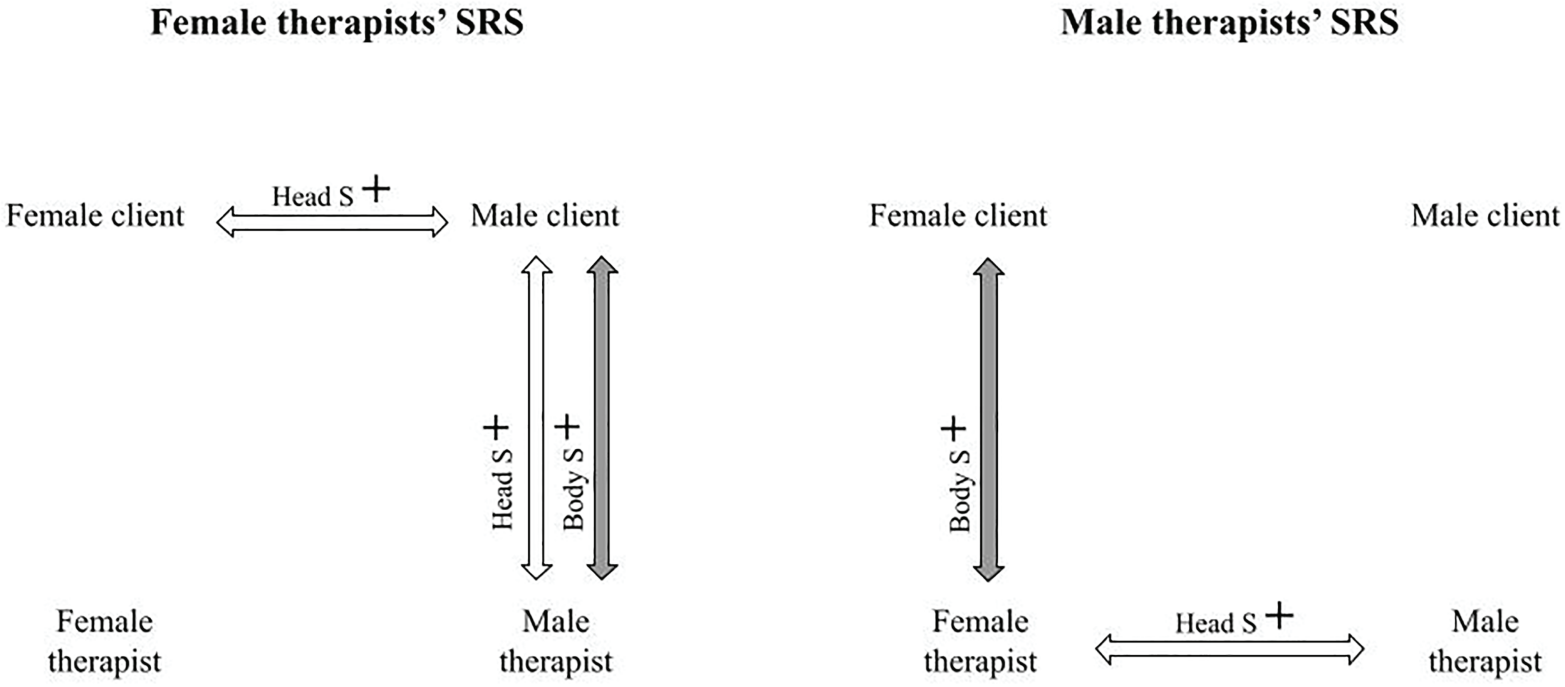
The relationship between the female and male therapists’ alliance evaluations and the dyadic synchronies.

The relationship between the participants’ evaluations of the alliance (SRS) and the head and body synchronies were calculated using complex models. The alliance was evaluated by all participants filling out the SRS after each session. All relations between the participants’ SRSs and the dyadic nonverbal synchronies are displayed in Table 7. The significant relations were quite evenly distributed among the different participants (female client had four significant relations, male client had two significant relations, female therapist had three significant relations, and male therapist had two significant relations). The relations between the participants’ evaluations of the alliance and the nonverbal synchrony patterns are illustrated in Figure 2 and Figure 3.

**Table 7:** The relations between all participants’ Session Rating Scale (SRS) evaluations and head and body synchronies (ES_noabs_).

Dyads	Client and client		Female client and male therapist		Female client and female therapist		Male client and male therapist		Male client and female therapist		Therapist and therapist	
	Head	Body	Head	Body	Head	Body	Head	Body	Head	Body	Head	Body
Female client SRS *β* (*p*)	−0.365^*^ (0.003)	−0.134 (0.296)	−0.009 (0.954)	0.324^*^ (0.040)	0.079(0.599)	−0.182 (0.306)	0.188 (0.257)	0.152 (0.297)	0.281 (0.086)	0.514^*^ (0.000)	0.178 (0.323)	0.481^*^ (0.000)
Male client SRS *β* (*p*)	−0.089 (0.733)	0.270 (0.180)	0.157 (0.351)	0.415^*^ (0.007)	0.268 (0.310)	0.073 (0.810)	0.244 (0.156)	0.136 (0.456)	0.451^*^ (0.022)	0.415 (0.065)	−0.0.057 (0.834)	0.283 (0.168)
Female therapist SRS *β* (*p*)	0.436^*^ (0.005)	0.077 (0.714)	−0.043 (0.725)	0.029 (0.748)	0.144 (0.555)	0.283 (0.226)	0.198^*^ (0.038)	0.449^*^ (0.002)	0.078 (0.707)	0.342 (0.147)	0.419 (0.062)	0.199 (0.348)
Male therapist SRS *β* (*p*)	−0.078 (0.505)	−0.148 (0.380)	0.045 (0.564)	−0.056 (0.674)	0.225 (0.103)	0.415^*^ (0.013)	0.044 (0.654)	0.262 (0.074)	0.013 (0.904)	0.163 (0.354)	0.304^*^ (0.002)	0.212 (0.075)

* Significant result.

The mean of both clients’ SRS evaluations was significantly related to the mean of all body synchronies (*β*=0.532, *p*<0.001), but not to the mean of all head synchronies (*β*=0.284, *p*=0.076). The mean of both therapists’ SRS was significantly related to both the mean of all head synchronies (*β*=0.305, *p*=0.005) and body synchronies (*β*=0.369, *p*=0.023).

For both clients, their SRS evaluations were related to the mean of all body synchronies in the sessions (female clients’ *β*=0.467, *p*=0.003; male clients’ *β*=0.449, *p*=0.012), but not to the mean of head synchronies (female clients’ *β*=0.158, *p*=0.371; male clients’ *β*=0.239, *p*=0.119). As for the therapists, the female therapists’ SRS evaluations were significantly related to the mean of both body (*β*=0.365, *p*=0.025) and head (*β*=0.316, *p*=0.004) synchrony. For the male therapists, no significant relations were found between their SRS evaluations and the mean of all body (*β*=0.198, *p*=0.121) or head synchronies (*β*=0.136, *p*=0.076).

As with ORS, we wanted to investigate whether there was any difference in the alliance evaluations regarding whether one participated in or observed nonverbal synchrony. The relationships were computed for each participant using complex models.

For female clients, their SRS was significantly related to the mean of the synchronies that they observed (*β*=0.315, *p*=0.046), but not to the synchronies in which they participated (*β*=0.043, *p*=0.831). For male clients, the opposite result was found: Their SRS was significantly related to synchronies in which they participated (*β*=0.341, *p*=0.027), but not to synchronies that they observed (*β*=0.329, *p*=0.066).

For female therapists, no significant relationships were found between their SRS evaluations and the synchronies in which they participated (*β*=0.259, *p*=0.291) or observed (*β*=0.269, *p*=0.051). The male therapists’ SRS evaluations were significantly related to the synchronies in which they participated (*β*=0.172, *p*=0.032) but not to the synchronies that they observed (*β*=0.193, *p*=0.101).

### Therapy Outcomes and Nonverbal Synchrony

The relation between CORE-OM and the dyadic nonverbal synchronies was calculated using data where the dyadic nonverbal synchronies were case wise aggregated, i.e., calculating the mean of head synchrony and the mean of body synchrony for each dyad in each case. Spearman’s rank order correlations were used. First, both client’s CORE-OM change scores were correlated with all dyadic nonverbal synchrony effect sizes. The female clients’ CORE-OM change scores from beginning to end were significantly related to body synchrony between the male client and female therapist *r*(4)=0.829, *p*=0.0041, and the female clients’ CORE-OM change scores from end to 6months were significantly related to head synchrony between the female client and male therapist *r*(4)=1.000. No significant relations between the male clients CORE-OM change scores and the dyadic nonverbal synchrony effect sizes were found.

Second, both clients’ CORE-OM change scores were correlated with the mean of all head and body synchronies. The male clients’ CORE-OM change score beginning to 6months was significantly correlated with the mean of all head synchronies *r*(4)=0.829, *p*=0.0042. No other significant correlations with the mean of head or body synchrony were found.

Third, the mean of both clients’ CORE-OM change scores was correlated with all dyadic head and body synchronies. The mean of both clients’ CORE-OM change score from the beginning to the end was significantly correlated with body synchrony between the male client and the female therapist *r*(4)=0.886, *p*=0.0019. No other significant correlations were found.

## Discussion

We explored whether nonverbal synchrony occurred in couple therapy, and if it was related to the well-being of the clients, and to the therapeutic alliance and therapy outcome. Nonverbal synchrony occurred above chance level in all sessions, and usually between all dyads. Importantly, significant nonverbal synchrony occurred between all possible dyads, meaning that all participants were included in the nonverbal synchronies. Nonsignificant synchrony was rare and occurred mostly between spouses (in three sessions, all in different cases). This was consistent with expectations, since nonverbal synchrony between spouses has been related to satisfaction in the relationship (Julien et al., 2000), and spouses coming to couple therapy have sought help because of difficulties in their relationship.

It is noteworthy that almost all of the synchronies showing in-phase, where both participants’ movements were positively correlated, were significant, as well as the majority of the anti-phase synchronies, where one participant moved more when the other one moved less (*cf*. Tschacher and Meier, 2020). Most of the few nonsignificant synchronies were anti-phase synchronies. Anti-phase synchrony could signal giving space to the other, that is, one person talking and nodding and the other listening, whereas in-phase synchrony could signal more of a mutual and more simultaneous involvement in the conversation. In our data, all head movement synchronies and almost all body movement synchronies between the co-therapists were in-phase synchrony, indicating that the therapists were moving more in unison.

Comparing the three different types of dyads (client–client, client–therapist, therapist–therapist) revealed that the co-therapists had indeed more head and body synchrony than the other kinds of dyads. Previous research on the same data also found a large amount of physiological synchrony (electrodermal activity) between co-therapists (Karvonen et al., 2016; Tourunen et al., 2020). The large amount of synchrony between the co-therapists can be interpreted as them being bodily involved similarly in the situation, in listening to the clients’ problems, and in trying to help them through their professional roles.

In the couple therapy it is important to detect both in-phase and anti-phase synchrony, since as multiple participants are present it makes the movement patterns more diverse. It is for instance usual that one dyad is talking and moving more, whereas the other dyad is listening and remaining quite still. Qualitative inspection of the couple therapy videos revealed that there could be long instances where one of the participants sat quite still listening to others talking. Thus, we assessed differences between how much the participants moved. Clients moved their heads and bodies more than therapists did, which is contrary to earlier findings in individual psychotherapy (Ramseyer and Tschacher, 2014). Female clients moved their heads more than male clients, which again replicated previous findings (Ramseyer and Tschacher, 2014). An opposite pattern was found for the therapists: male therapists moved their heads more than female therapists. Head movements were usually speech-related, that is, nodding while talking or listening, or signal turn-taking. Nods have been reported as signs of affiliation (Stivers, 2008). It would be interesting to study whether more head movements in this data were related to talking more in the session. The fact that clients moved more than therapists could be related to the couple therapy context, where the clients’ lives and their relationship form the content of the session, whereas the couple therapists’ main function is to be receptive, listen to the clients, and help them.

As the data originated from a research project studying the participants’ autonomous nervous system responses, the participants wore autonomous nervous system equipment in some of the sessions. We wanted to know if these altered the movement patterns of the participants. No differences were found for individual movements. For nonverbal synchrony, the only difference was that there was less head movement synchrony between the therapists in the measurement sessions. All of the therapists’ head synchronies were in-phase synchronies, meaning that they both moved their head more (or less) when synchrony occurred. It might be that wearing measurement equipment made the situation novel even for the therapists and made them concentrate more on their own thoughts and bodily reactions, thus affecting how they worked together but not how they nonverbally related to the clients.

### Clients’ Well-Being and Nonverbal Synchrony

The clients evaluated their well-being with ORS at the beginning of each session (the recommended use of ORS; *cf*. Anker et al., 2010; Kuhlman et al., 2013), which makes it possible to establish how their well-being affected their participation in the nonverbal synchronies in the session. The well-being of the clients was related to the mean of all body but not head synchronies, which means that when clients felt better, there was more body movement synchrony between all participants. Bodily movements are more unspecific than head movements and can be speech-related gesturing or shifting postures. It has been suggested that body movements are more implicit than head movements, and thus could be related to the immediate situation and emotions within it (Ramseyer and Tschacher, 2014).

The well-being of the clients was not related to them participating in or observing nonverbal synchrony. Even though we were not interested in gender differences *per se*, gender differences were found. As the female client felt better, there was more head and body synchrony between the male client and the female therapist, and body synchrony between herself and the male therapist. The female clients’ well-being has also been related to physiological synchrony between male client and female therapist (Tourunen et al., 2020). When the male client felt better at the beginning of the session, there was more body synchrony between himself and the female therapist and more head and body synchrony between the therapists. The fact that synchrony between the therapists was related to the male clients’ well-being suggests that it was as if the therapists implicitly adjusted their co-working style according to how the male client was feeling. Interestingly when the clients felt better, they were more bodily synchronized with the therapist of the opposite gender.

### Therapeutic Alliance and Nonverbal Synchrony

Nonverbal synchrony has been suggested as a marker of therapeutic alliance in individual psychotherapy (Koole and Tschacher, 2016). In couple therapy the context is more complex, and we wanted to explore the relationship between nonverbal synchrony and the therapeutic alliance. The alliance was evaluated after each session; therefore, it can be interpreted as being associated with the nonverbal synchrony patterns occurring earlier in the session. Associations were found between the nonverbal synchrony patterns and the therapeutic alliance, even in this small data set. In accordance with previous research (Ramseyer and Tschacher, 2014) we found that clients evaluated the alliance as stronger when there was more body synchrony in the session. For the therapists, both head and body synchrony were related to their evaluations of the alliance, which has not been reported before.

Interestingly, female clients evaluated the alliance to be stronger when they observed more synchrony between the other participants in the session (in contrast to taking part in synchronies themselves), whereas male clients and male therapists evaluated the alliance to be stronger when they participated in nonverbal synchronies. For female therapists, no such associations were found. It appears possible that when the female clients saw other persons synchronizing together, they implicitly felt that something important was being worked on. Research on physiological synchrony using the same data revealed that female clients also made increasingly better evaluations of the alliance when the physiological synchrony between their spouse and the male therapist increased (Tourunen et al., 2020).

A detailed examination of the relation between nonverbal synchrony and alliance revealed that for both female and male clients’ alliance evaluations, synchrony between male client and female therapist, and female client and male therapist, were of importance. It is interesting that synchrony between the same dyads were relevant for clients of both genders. It seems that both clients implicitly felt the importance of all participants being included in the dyadic synchrony patterns for them to evaluate the working relationship to be of good quality. This pattern of dyadic synchrony between the opposite genders was not seen in the psychotherapists’ alliance evaluations. On the contrary, both therapists evaluated the alliance to be stronger when there was more synchrony between the two participants of the same gender but opposite to themselves (the other therapist and one of the spouses). For female therapists, head and body synchrony between the male client and male therapist were significant. For the male therapists, body synchrony between the female client and female therapist was significant. Head synchrony between the co-therapists was related to the male therapists’ but not the female therapists’ evaluations of the alliance. We found such a difference interesting, as it could suggest that female and male therapists implicitly concentrated on different aspects of co-working. It might not be gender related but having to do with the male therapists being more experienced family therapists than the female therapists.

For female therapists, more head synchrony between clients was related to them evaluating the alliance to be stronger. It might be that the female therapists implicitly evaluated synchrony between clients as a positive marker of their relationship as previous research has suggested (Julien et al., 2000; Sharon-David et al., 2019). But surprisingly, more head synchrony between the spouses made the female clients evaluate the alliance to be weaker. Head movement synchrony might signal active participation in the conversation, nodding together could signal agreement on the subject. Agreement, if too early in the therapy process, could create an impasse, and might make it difficult to bring forth difficult subjects concerning the relationship. Nonverbal synchrony has previously been related also to negative aspects of the relationship, such as blurring boundaries between people (Paladino et al., 2010) or negatively affecting self-regulation (Galbusera et al., 2019). It is important to recognize that nonverbal synchrony might not always serve a good purpose in couple therapy.

Even though the results have been presented by gender, it is important to keep in mind that as the data were quite small, generalizations based on the gender cannot be made. The gender was needed in the statistical computations to distinguish between the two therapists and the two clients. To summarize, the significant nonverbal patterns for the alliance differed between the therapists and clients, suggesting that they implicitly experienced different nonverbal synchrony patterns as relevant. For clients, nonverbal synchrony by dyads of the opposite gender was related to their alliance evaluations, whereas for the therapists, nonverbal synchrony by dyads of the same gender but opposite to their own were related to their evaluations of the alliance.

### Therapy Outcome and Nonverbal Synchrony

The relationship between the CORE-OM outcome measurement and nonverbal synchronies must be considered precursory, since the data set was extremely small. It is noteworthy that some of the relationships between the outcome change scores and nonverbal synchrony were similar to the relationships between the therapeutic alliance evaluations and nonverbal synchrony. For instance, for female client, changes from the beginning of therapy to the end of therapy were related to body synchrony between the male client and the female therapist, and her six-month follow-up was related to head synchrony between herself and the male therapist. These same dyads were related to her alliance evaluations. For male clients, head synchrony among all participants was related to his outcome 6months after the therapy ended.

### Conclusion

The study presented here is the first to study nonverbal synchrony in couple therapy. In spite of the small amount of data, nonverbal synchrony was significant between the majority of dyads, and we found significant relations between nonverbal synchrony and the clients’ well-being, alliance, and therapy outcome.

One important finding was the difference between therapists and clients, concerning which dyads were related to their alliance evaluations. For clients, synchrony between dyads of the opposite gender was relevant, and that all participants were included in the nonverbal synchronies. In particular, synchrony between the male client and female therapist was related to both clients’ well-being, to both clients’ evaluations of the alliance, and to the therapy outcome for the clients. For therapists, other patterns were found, such as synchrony in same-gender dyads relating to their alliance evaluations. The results were reported gender-wise to distinguish between the four participants, but other unknown variables could lie behind the associations.

Our findings suggest that nonverbal synchrony is a potential marker of therapeutic alliance in couple therapy, albeit with some restrictions. The relationship between nonverbal synchrony, alliance, and outcome is more complex in a multi-actor context, where there are multiple relationships and alliances at play compared to individual psychotherapy. The couple therapy conducted in our data was not manualized, and the therapists used dialogical and system-therapeutic ways of working. The fact that there was nonverbal synchrony in all sessions and among almost all dyads is in line with earlier research that have demonstrated nonverbal synchrony during interactions and in the therapeutic context, especially in non-manualized therapies (Altmann et al., 2020). But the presence of four participants with different roles within the situation made the context more complex.

### Clinical Implications

Nonverbal synchrony can be seen as a process variable influencing the outcome of therapy (Prinz et al., 2021), as nonverbal synchrony could serve important functions in couple therapy, signalling attunement (Stel and Vonk, 2010), empathy (Finset and Ørnes, 2017), and helping to connect with others (Miles et al., 2010). Thus, nonverbal synchrony is a vital part of therapy because it enables participants to feel connected to and understood by others. But nonverbal synchrony could have other functions as well. Research on interaction (not in the context of psychotherapy) has suggested that nonverbal synchrony could serve a compensatory function, smoothing out the interaction when there is a lack of synchrony in some other aspect of the interaction (Dale et al., 2020). This corresponds to previous research reporting that verbal and nonverbal markers of alliance were not always in congruence in couple therapy (Kykyri et al., 2019).

Our results indicate that the relation between nonverbal synchrony and alliance and outcome in couple therapy is not straightforward but affects spouses and therapists (and even female and male participants) in different ways. It is crucial for the therapist to be attentive to the nonverbal synchrony patterns in the sessions since they can be related to the well-being of the clients, to therapeutic alliance, and even outcome. But conclusions cannot be made based on this study alone, more research is needed.

There is for instance some evidence from research on individual psychotherapy that a curvilinear model of nonverbal synchrony would be best, where a medium-level synchrony may offer better outcomes than low or high synchrony (Paulick et al., 2018a). Research on mother–infant synchrony has given similar suggestions. A high amount of synchrony is not always beneficial for the developing child, but it might lead to a more insecure attachment (Beebe and Steele, 2013). On the other hand, persons with secure attachments have a tendency to synchronize less to others (Feniger-Schaal et al., 2016). Intimate relationships and crises within them bring forth the individual’s attachment style, and manifest in couple therapy. More research is needed in the couple therapy context to be able to discover if these findings apply to couple therapy as well.

The result that synchrony of head movements between the spouses was negatively related to the female clients’ alliance evaluations suggests that nonverbal synchrony might not always serve a good purpose in couple therapy. Previous research has also hinted that it is equally important to be able to withdraw from synchronizing with others in some situations. For clients, not synchronizing with others might help in the self-regulation of affect (Galbusera et al., 2019). For therapists synchronizing with the client might in some situations reinforce the client’s negative behaviors (Bänninger-Huber and Widmer, 1999; Lutz et al., 2020). Mayo and Gordon (2020) suggested that it is important to study moving in and out of synchrony, since there are always two forces working simultaneously: adjusting and synchronizing to others as well as withdrawing from synchrony and acting independently.

### Limitations and Suggestions for Future Research

The dyadic nonverbal synchrony data were based on 29 sessions, and it comprised data from only 11 couple therapy cases, thus generalizations cannot be made. Because of the limited number of cases, we obtained nonverbal synchrony from the entire therapy sessions, something that is not common. Nevertheless, the results from this study should be regarded as exploratory. The presence of four participants resulted in six different dyads that could synchronize in either head or body movements. Dyadic synchrony is the most common form of nonverbal synchrony studied in psychotherapy settings. Couple therapy with four participants would provide a good context for studying triadic and quadratic synchrony, which was not done in the study presented here. In the future, the procedure of calculating triadic synchrony (Dale et al., 2020) or the multivariate synchrony approach (Galbusera et al., 2019; Meier and Tschacher, 2021) could be used. It would be interesting to find out the extent to which triadic or generally multivariate synchrony occurs in couple therapy.

Another shortcoming of the study presented here is that it was not possible to use multilevel modeling because of the small amount of data within clusters. The statistical methods chosen were the best option for overcoming this difficulty.

The use of self-report measures for the evaluation of subjective well-being, the therapeutic alliance, and outcome can be criticized. In particular, the ultra-brief forms of ORS and SRS could be criticized for not giving a detailed enough account of the evaluations. For instance, the alliance was evaluated holistically by the participants, meaning that both spouses evaluated the therapists as a team, and the therapists evaluated the alliance to both spouses simultaneously. However, the use of ultra-brief forms is clinically sound, since filling out the forms is convenient, even in a standard therapy setting. The large amount of missing data in the CORE-OM form was unfortunate, and the results should be read with caution.

In the future, research with a larger dataset is needed to confirm the results. It would also be interesting to study what variables affect nonverbal synchrony in the couple therapy context. For example, does the content of the discussion affect the synchrony patterns? What variables induce in-phase or anti-phase synchrony? Do the synchrony patterns reveal the participants’ views or attitudes towards the topic spoken of? Does nonverbal synchrony signal like-mindedness or empathy in the couple therapy context? It would also be interesting to further elaborate on the relationship between nonverbal synchrony and alliance based on the In-sync model (Koole and Tschacher, 2016). An alliance measure that would be better suited to the couple therapy context would be beneficial to help study the relationship between systemic alliances and nonverbal synchronies. Could nonverbal synchrony be used to study alliance ruptures, as Friedman (2020) suggested? We suggest that more research on nonverbal synchrony in couple therapy is needed, since nonverbal synchrony could be used as a marker of therapeutic alliance and could be seen as reflecting the clients’ well-being.

## Data Availability Statement

The datasets presented in this article are not readily available because the data is confidential psychotherapy data from 11 couple therapy cases, with data organized by gender. Even though the data is anonymized, because of the small *N*, we are not comfortable with sharing it openly. Requests to access the datasets should be directed to PN-S, petra.nyman-salonen@jyu.fi.

## Ethics Statement

The studies involving human participants were reviewed and approved by University of Jyväskylä Ethical Committee. The patients/participants provided their written informed consent to participate in this study.

## Author Contributions

PN-S: first authorship. V-LK: senior authorship, research design, and collecting of the data. WT: senior authorship and synchrony computation. JM: statistical methods. AT: research design and collecting of the data. MP: research design and collecting of the data. JS: last authorship, research design, and collecting of the data. All authors contributed to the article and approved the submitted version.

## Funding

Academy of Finland, grant number 265492 (Relational Mind in Events of Change in Multiactor Therapeutic Dialogues). Kone foundation, grant number 201710524 (Experiential Demarcation: Multidisciplinary Inquiries into the Affective Foundations of Interaction).

## Conflict of Interest

The authors declare that the research was conducted in the absence of any commercial or financial relationships that could be construed as a potential conflict of interest.

## Publisher’s Note

All claims expressed in this article are solely those of the authors and do not necessarily represent those of their affiliated organizations, or those of the publisher, the editors and the reviewers. Any product that may be evaluated in this article, or claim that may be made by its manufacturer, is not guaranteed or endorsed by the publisher.

## Footnotes

^1^The reason for the measurement videos being of better quality was that they were adjusted at the beginning of the session by a researcher, and were monitored throughout to ensure the quality of the video, whereas the videos from the regular sessions were recorded by the therapists, who put the recording on at the beginning of the sessions, and the videos were not monitored for quality during the sessions. This resulted in inadequate zoomings, that is, the whole bodies of one or two participants were not visible on the screen.
